# Validation of the brief scale for the evaluation of acculturation stress in migrant population (EBEA)

**DOI:** 10.1186/s41155-020-00168-3

**Published:** 2021-01-06

**Authors:** Alfonso Urzúa, Diego Henríquez, Alejandra Caqueo-Urízar, Vanessa Smith-Castro

**Affiliations:** 1 Escuela de Psicología, Universidad Católica del Norte, Avda. Angamos, 0610 Antofagasta, Chile; 2 Instituto de Alta investigación, Universidad de Tarapacá, Arica, Chile; 3 Instituto de Investigaciones Psicológicas, Universidad de Costa Rica, San José, Costa Rica

**Keywords:** Acculturation, Acculturation stress, Migrant, Migration, Stress

## Abstract

**Background:**

Acculturation stress is associated with poorer physical and mental health and a lower level of psychological well-being. The causes of acculturation stress are diverse, but most are similar in the migrant population. Despite the importance of evaluating this variable, few studies have reported culturally adapted and validated instruments for specific populations. Based on this, the aim of this study was to evaluate the psychometric properties of a short scale for the evaluation of acculturation stress (EBEA).

**Methods:**

Two studies were conducted, involving 1725 first-generation Colombian and Peruvian migrants living in Chile, between the ages of 18 and 60 years. In addition to the EBEA and as evidence of validity, the Beck Anxiety Scale and the WHOQOL-BREF psychological health domain were applied. A confirmatory factor analysis was carried out, and the reliability and nomological validity were evaluated.

**Results:**

The results in both studies indicated that the scale presents a factorial structure of three dimensions: (a) the stress derived from the preparation and departure from the country of origin, (b) the stress produced by socioeconomic concerns in the host country, and (c) the tensions typical of adaptation to sociocultural changes or Chilean society. The reliability coefficients and the analysis of their nomological validity were very good.

**Conclusions:**

The EBEA is a measure that offers quick, useful screening for researchers who need a short measure for research among migrants. This tool contributes to the work of education, prevention, and intervention in the field of general health and migrants’ mental health.

## Introduction

By mid-2019, about 272 million people were living outside their country of birth (United Nations, 2019), of which 10 million were immigrants living in South America (International Organization for Migration, 2020). In addition, migration pressures within and outside borders are expected to increase in the coming decades due to demographic forces, changes in the labour market, and climate change (World Bank Group, 2020).

From a psychological perspective, migration implies major changes to people’s lives, often accompanied by distinctive stressors that affect their psychological well-being and adaptation (Berry, 2008). Stress by acculturation is a concept coined by the psychology of intercultural contact and acculturation to describe this specific type of stress (Berry, Kim, Minde, & Mok, 1987). According to the literature, acculturation is understood as a process resulting from contact between two or more cultural groups with impacts at a group level, producing transformations in social and institutional structures, and at the individual level, bringing about behavioural changes (Berry, 2005).

People involved in the process of acculturation experience processes of change in different areas of psychological functioning, such as language, cognitive styles, personal and social identity, attitudes and values, and behaviour. These processes of change can evolve with great fluidity and ease, but can also be more problematic, accompanied by cultural conflict and inter-group tensions (Berry, 2005, 2008).

At the individual level, such changes may be expressed as simple behavioural adaptations (modifications in ways of dressing, speaking, eating), or may become more complicated and be reflected in states of stress and manifestations such as uncertainty, anxiety, or depression (Berry, 2005). Specifically, when the demands of adaptation to the new culture exceed people’s abilities to cope, this type of stress arises, which is defined as the experience of adverse physical and emotional reactions as a result of the complex process of adaptation to a new cultural context (Arbona et al., 2010).

Indeed, previous studies have found important associations between acculturation stress and physical and mental health problems in adults (Sternberg & Lee, 2013; Urzúa, Basabe, Pizarro, & Ferrer, 2017; Urzúa, Heredia, & Caqueo-Urízar, 2016), adolescents (Belhadj and Koglin, 2015), and children (Urzúa, Caqueo-Urízar, & Flores, 2019). Moreover, it occurs in various sociocultural groups involved in acculturation processes such as refugees (Ugalde-Watson, Smith-Castro, Moreno-Salas, & Rodríguez-García, 2011), immigrants (Mera-Lemp, Martínez-Zelaya, Orellana, & Smith-Castro, 2020; Urzúa, Caqueo-Urízar, Carvajal, & Páez, 2017; Urzúa, Leiva-Gutiérrez, Caqueo-Urízar, & Vera-Villarroel, 2019), international students (Castro and Perugini, 2013), and ethnic minorities (Smith-Castro, 2003).

Within this research corpus, important efforts have been dedicated to measuring acculturation stress in an adequate manner, either through general measures of stress, anxiety, depression, life satisfaction, self-esteem, and other behavioural indicators, or through the construction of instruments to measure this specific type of stress (Bashir & Khalid, 2020; Castro-Olivo, Palardy, Albeg, & Williamson, 2014; Chavez, Moran, Reid, & Lopez, 1997; Rudmin, 2009). In Latin America, there is a history of instruments used to measure this type of stress in refugees (Ugalde-Watson et al., 2011) and immigrant children (Urzúa, Caqueo-Urízar, & Flores, 2019). However, these measures have been constructed specifically for such populations, so their usefulness in applying them to other groups is limited.

The aim of this paper is to be the first to confirm, through two studies, the validity and internal consistency of the brief scale to evaluate acculturation stress in a migrant population. The Brief Scale of Acculturation Stress (Escala Breve para la Evaluación del Estrés por Aculturación, EBEA) was constructed to measure three dimensions of the degree of stress perceived in the migratory process, namely (1) the stress derived from preparation and departure from the country of origin, (2) the stress produced by socioeconomic concerns in the host country, and (3) the tensions inherent to adaption to sociocultural changes or Chilean society. These dimensions cover relevant aspects of the experiences of immigrants once they have settled in the receiving society and aspects of the emigration process that are not covered by other measures available in our environment.

It is expected that EBEA will be useful to execute a rapid screening of the effects of migration on the immigrant population in general, which in turn can serve for future research in the area, constituting a tool that contributes to the work of education, prevention, and intervention in the field of general health and mental health with these groups.

## Methods

### Design

The study had a cross-sectional and instrumental design. Convenience sampling was used.

### Sample

#### Study 1

Of the 912 Colombian immigrants, 51.9% lived in Antofagasta (*n* = 473), 23.7% in Arica (*n* = 216), and 24.5% in Santiago (*n* = 223), Chile. Participants’ ages ranged from 18 to 60 years, with a mean age of 35.06 years (SD = 9.65). A total of 461 participants were women (50.5%) and 451 participants were men (49.5%). The participants agreed voluntarily and anonymously to complete the questionnaire after signing an informed consent form.

#### Study 2

A total of 831 immigrants participated, of whom 52.2% (*n* = 434) were Colombians (sample 1) and 47.8% were Peruvians (sample 2). Among sample 1, 53.2% resided in Antofagasta (*n* = 231), 23.3% in Arica (*n* = 101), and 23.5% in Santiago (*n* = 102), Chile. The age of the participants ranged from 18 to 60 years, with a mean age of 32.61 years (SD = 8.96). A total of 229 participants were women (52.8%) and 205 participants were men (47.2%). As for sample 2, 49.4% resided in Antofagasta (*n* = 196), 24.9% in Arica (*n* = 99), and 25.7% in Santiago (*n* = 102), Chile. Participants’ ages ranged from 18 to 60 years, with a mean age of 33.35 years (SD = 9.40). A total of 198 participants were women (49.9%) and 199 participants were men (50.1%). Table 1 presents the summary and some additional characteristics of participants in both studies. The participants agreed voluntarily and anonymously to fill out the questionnaire after signing an informed consent form.

**Table 1:** Participants and characteristics

Variable	Category	Study 1	Study 2		Total (*n* = 1743), *n* (%)
		Colombian (*n* = 912), *n* (%)	Colombian (*n* = 434), *n* (%)	Peruvian (*n* = 397), *n* (%)	
Gender	Male	451 (49.5)	205 (47.2)	199 (50.1)	855 (49.1)
	Female	461 (50.5)	229 (52.8)	198 (49.9)	888 (50.9)
Age group	18–30	319 (35.0)	213 (49.1)	170 (42.8)	702 (40.3)
	31–60	593 (65.0)	221 (50.9)	227 (57.2)	1041 (59.7)
Arrival year	2000–2009	40 (4.5)	49 (6.0)	149 (38.3)	238 (14.0)
	2010–2019	848 (95.5)	380 (46.5)	240 (29.3)	1468 (86.0)
City	Northern end (Arica)	216 (23.7)	101 (23.3)	99 (24.9)	416 (23.9)
	North (Antofagasta)	473 (51.9)	231 (53.2)	196 (49.4)	900 (51.6)
	Centre (Santiago)	223 (24.5)	102 (23.5)	102 (25.7)	427 (24.5)
Years of education	Less than 8 years of education	101 (11. 2)	26 (3.2)	33 (4.1)	160 (9.3)
	8 years of education	230 (25.6)	89 (10.9)	69 (8.5)	388 (22.7)
	12 years of education	309 (34.4)	144 (17.7)	129 (15.8)	582 (34.0)
	More than 12 years of education	258 (28.7)	161 (19.8)	163 (20.0)	582 (34.0)
Occupational situation	Active worker	653 (75.3)	300 (38.2)	289 (36.8)	1242 (75.2)
	Unemployed	121 (14.0)	67 (8.5)	49 (6.2)	237 (14.3)
	Student	33 (3.8)	14 (1.8)	8 (1.0)	55 (3.3)
	Homemaker	57 (6.6)	25 (3.2)	27 (3.4)	109 (6.6)
	Retired or pensioned	3 (0.3)	6 (0.8)	0 (0.0)	9 (0.5)

### Measures

#### Acculturation stress

The Brief Scale for the Evaluation of Acculturation Stress (Escala Breve para la Evaluación del Estrés por Aculturación, EBEA), designed for this study, assesses three dimensions of the degree of stress perceived in the migration process: (1) preparation and departure from the country of origin (six items), (2) socioeconomic concerns (four items), and (3) adaptation to the receiving society (four items). The original scale is made up of 33 items specifically designed to measure acculturation stress among the refugee population in Costa Rica (Ugalde-Watson et al., 2011). Items ask participants to rate the degree of stress (fear or anxiety) experimented through distinct phases of their migration process, from the decision to leave Colombia to the arrival and settlement in Costa Rica. A preliminary version of the scale was peer-reviewed by social and cross-cultural psychologists in Costa Rica. Cognitive interviewing (Smith-Castro and Molina, 2011; Willis, 2005) was employed to detect comprehension problems. Specifically, two female and two male refugees completed the questionnaire and reported cognitive challenges in answering the items. The definitive version of the scale was applied to 100 refugees from Colombia (57% women), with ages ranging from 18 to 68 years (M = 39.68 years, SD = 11.93 years), who had been living in Costa Rica around 4 years (M = 4.14, SD = 1.30) (Ugalde-Watson et al., 2011).

In this research, we used a reduced version of the original scale, which was developed in a previous study on discrimination, stress, and well-being through structural equation models. The original version was adapted using information obtained through cognitive interviews with Colombian and Peruvian migrants. Furthermore, with the authorization of the authors, the refugee dimension was eliminated. The scale measurement models were analysed prior to the analysis of structural equations, which resulted in the abbreviated scale used in the present investigation as a result.

The EBEA items are presented under the slogan ‘How stressful (tense or distressing) did you find it...’, for example, ‘Adapting to Chilean ways of speaking’. The answer options were based on a 5-point Likert scale, responses ranged from 1 ‘Not at all stressful’ to 5 ‘Very stressful’. High scores would account for a higher degree of acculturation stress.

#### Beck Anxiety Inventory

The Beck Anxiety Inventory (BAI) is a questionnaire that assesses common symptoms associated with anxiety disorders via 21 questions. Each item is scored from 0 to 3 points, where the higher the score, the greater the presence of anxious symptoms. In this study, the Spanish version (Magán, Sanz, & García-Vera, 2008; Sanz, 2014) of the questionnaire elaborated in 1988 (Beck, Epstein, Brown, & Steer, 1988) was used. This scale presented adequate levels of reliability (α = .96; ω = .96), and its measurement model was adequately adjusted to the data (Par = 84; *χ*^2^ = 1352.927; DF = 189; *p* = .00; IFC = .969; TLI = .966; RMSEA = .08).

#### Psychological health (SPS)

To assess this, the domain on psychological health of the WHOQOL-BREF questionnaire was used (World Health Organization Quality of Life Group - WhoQoL Group, 1998); it was translated and adapted to Spanish by Lucas-Carrasco (Lucas-Carrasco, 1998; Lucas-Carrasco, 2012). This questionnaire has presented valid and reliable scores in the Chilean context (Benitez-Borrego, Guardia-Olmos, & Urzúa-Morales, 2014; Benítez-Borrego, Mancho-Fora, Farràs-Permanyer, Urzúa-Morales, & Guàrdia-Olmos, 2016; Urzúa & Caqueo-Urízar, 2013) and has already been used in an immigrant population (Urzúa et al., 2015; Urzúa et al., 2017).

The complete questionnaire is structured around 26 questions grouped into four domains: physical, psychological, environmental, and social, of which we have used only the psychological one given its relationship with the variable under study. This domain contains six items that reflect various facets of psychological health: positive feelings (‘How much do you enjoy life?’), personal beliefs (‘How much do you feel your life has meaning?’), concentration (‘What is your ability to concentrate?’), body image (‘Are you able to accept your physical appearance?’), self-esteem (‘How satisfied are you with yourself?’), and negative feelings (‘How often did you have negative feelings such as sadness, hopelessness, anxiety, and depression?’). The answers are given on a Likert-type scale, with options ranging from 1 to 5. The higher the score, the better the person’s psychological health. In our study, the scale scores presented good reliability (α = .71; ω = .72), and its measurement model was adequately adjusted to the data (Par = 30; *χ*^2^ = 27,874; DF = 9; *p* = .00; IFC = .988; TLI = .980; RMSEA = .05).

Acculturation stress was measured in both studies, but since there are different studies, anxiety (BAI) was only measured in study 1 and psychological health (SPS) was only measured in study 2.

### Procedures

This research is part of a larger project that assesses the effect of discrimination on the health and well-being of Chile’s immigrant population, which was reviewed and approved by the ethics committee of the Universidad Católica del Norte, Chile. Before applying the measurement instruments, respondents were asked to sign an informed consent form authorising the use of their answers for research purposes. Once the consent was signed, it was kept in a sealed envelope, to ensure the anonymity of the person.

In both studies, the questionnaires were anonymous and confidential, and were distributed in places with an influx of foreigners such as the Department of Immigration, the Jesuit Migrant Service, and other areas where immigrant populations congregate in the cities of Arica, Antofagasta, and Santiago, Chile. Each questionnaire was answered individually in the presence of a surveyor to resolve any doubts regarding the understanding of the instruments. The interviewers were undergraduate students working on a thesis who were specifically trained in the application of the instrument.

### Statistical analysis

The database was analysed using the statistical software SPSS 24 and Mplus 8.2. First, to provide evidence of the factor structure of the test, a measurement model was estimated by confirmatory factor analysis on the samples of the two studies separately. The model had to reflect the three theoretical dimensions of the construct by obtaining high factorial saturations (λ > .5) of the items for each of its factors: (1) preparation and departure from the country of origin (6 items), (2) socioeconomic concerns (4 items), and (3) adaptation to the recipient society (4 items). Alternatively, a one-dimensional model was also evaluated, to rule out the possibility that the EBEA could reflect a single general factor.

Second, to provide evidence of the reliability of the test scores, internal consistency was estimated by calculating Cronbach’s alpha coefficient (α) and omega coefficient (ω) (Revelle & Zinbarg, 2009) for each of the scale dimensions in each sample.

Third, to assess the equivalence of the scale between different groups, two factorial invariance tests were performed, using gender and home country as grouping variables. Following the recommendations of Chen (2007), differences in fit between the levels of configural invariance (as a base model), metric, and scalar were analysed. Changes below .010 for CFI and .015 for RMSEA are indicative of factor invariance between groups (Chen, 2007).

Finally, nomological validity was evaluated, which refers to the existence of the empirical relationships between hypothetically related constructs (Aldás Manzano & Uriel Jimenez, 2017). To provide evidence of the EBEA’s nomological validity, three structural equation models were estimated. The first model (M1) presents the relation between the scores of the dimensions of the EBEA and the BAI (study 1). The second and third models (M2 and M3) present the relation between the scores of the EBEA and the psychological health in two different samples (study 2: sample 1 = Colombians, sample 2 = Peruvians).

For the analysis of the models, the method of robust weighted least square mean and variance (WLSMV) was used, which is robust with non-normal ordinal variables (Beauducel & Herzberg, 2006). Model fit was evaluated using several indexes: the comparative fit index (CFI), the Tucker-Lewis Index (TLI), the root mean square error of approximation (RMSEA), and the chi-square (*χ*^2^). These indexes provide information about the discrepancy between the variance/covariance matrix proposed by the theoretical model (the proposed factor structure) and the variance/covariance matrix provided by the subjects (Hu & Bentler, 1995). In general, a model is said to fit the data acceptably if the IFC and TLI are greater than .90, the RMSEA is equal to or less than .05, and the chi-square value is low and not significant (Schreiber, 2017). Because *χ*^2^ is usually significant in large samples such as these, even when the models are properly adjusted (Hu & Bentler, 1995), this index is interpreted with caution.

## Results

### Descriptive information of the measurement instruments

Table 2 presents information on the means and standard deviations of the EBEA items and the mean BAI and SPS scores separated by sex in each sample.

**Table 2:** Descriptive information of the measurement instruments

Brief Acculturation Stress Scale (EBEA)	E1 COL		E2 COL		E2 PER	
	M, *ME (SD)*	F, *ME (SD)*	M, *ME (SD)*	F, *ME (SD)*	M, *ME (SD)*	F, *ME (SD)*
Preparation and departure from country of origin (PSP)	3.33 (1.02)	3.49 (1.02)	3.26 (1.16)	3.41 (1.15)	2.93 (1.12)	3.53 (1.04)
1. Prepare to leave your country.	3.21 (1.31)	3.30 (1.29)	3.08 (1.46)	3.26 (1.53)	2.72 (1.37)	3.40 (1.42)
2. Get the money to leave.	3.16 (1.36)	3.40 (1.36)	3.17 (1.58)	3.21 (1.57)	2.76 (1.46)	3.26 (1.47)
3. Leave your country.	3.31 (1.35)	3.50 (1.34)	3.09 (1.56)	3.25 (1.58)	2.84 (1.37)	3.54 (1.39)
4. Moving from your country to Chile.	3.35 (1.27)	3.44 (1.34)	3.22 (1.58)	3.34 (1.54)	3.08 (1.47)	3.48 (1.33)
5. Having to be separated from your family.	3.64 (1.26)	3.86 (1.30)	3.81 (1.36)	4.08 (1.31)	3.29 (1.53)	3.93 (1.23)
6. Stop seeing friends.	3.23 (1.29)	3.23 (1.37)	3.19 (1.48)	3.45 (1.49)	2.89 (1.32)	3.48 (1.33)
Socioeconomic concerns (PES)	3.32 (1.12)	3.42 (1.15)	3.45 (1.18)	3.49 (1.21)	2.99 (1.17)	3.61 (1.18)
1. Cover your basic needs.	3.33 (1.26)	3.44 (1.33)	3.66 (1.37)	3.56 (1.38)	3.12 (1.39)	3.70 (1.28)
2. Find a place to live.	3.15 (1.28)	3.28 (1.37)	3.33 (1.52)	3.42 (1.48)	2.94 (1.41)	3.52 (1.39)
3. Find a job.	3.35 (1.32)	3.42 (1.34)	3.33 (1.43)	3.42 (1.42)	2.92 (1.43)	3.44 (1.44)
4. Stabilise economically.	3.37 (1.26)	3.49 (1.26)	3.55 (1.35)	3.51 (1.40)	3.11 (1.27)	3.70 (1.37)
Adaptation to the recipient society (ASR)	2.42 (1.16)	2.37 (1.21)	2.11 (1.03)	2.10 (1.06)	2.28 (1.15)	2.37 (1.03)
1. Adapt to Chilean’s way of speaking.	2.59 (1.34)	2.66 (1.38)	2.31 (1.32)	2.33 (1.34)	2.34 (1.31)	2.51 (1.40)
2. Initiate contact with the neighbours.	2.43 (1.29)	2.37 (1.38)	2.23 (1.32)	2.16 (1.34)	2.25 (1.26)	2.43 (1.31)
3. Make friends.	2.41 (1.29)	2.29 (1.36)	2.09 (1.29)	1.96 (1.26)	2.29 (1.29)	2.27 (1.19)
4. Establish relationships with the people you work with.	2.33 (1.30)	2.17 (1.33)	2.06 (1.28)	1.88 (1.27)	2.22 (1.36)	2.19 (1.26)
**Other measures**						
Beck’s Anxiety Inventory (BAI)	0.50 (0.57)	0.47 (0.53)				
Psychological health (SPS)			3.67 (0.60)	3.57 (0.60)	3.42 (0.54)	3.26 (0.55)

*E1* study 1, *E2* study 2, *COL* Colombian sample, *PER* Peruvian sample, *M* male, *F* female, *ME* mean, *SD* standard deviation, *EBEA* Acculturation Stress Brief Scale, *PSP* preparation and departure from the country of origin, *PSE* socioeconomic concerns, *ASR* adaptation to the receiving society

### Factorial structure and estimated reliability

Table 3 shows the EBEA goodness-of-fit indicators in both studies and for each model. As can be seen, we found no evidence to support the plausibility of EBEA representing a one-dimensional structure of acculturation stress. On the contrary, the results of the models of measurement of three factors of the EBEA presented adequate indexes of goodness of adjustment, close to those recommended by the literature, in all the samples (Schreiber, 2017).

**Table 3:** Global adjustment indicators of the EBEA measurement models

Number of factors	Study and sample	Par	*χ*^2^	*DF*	*p*	CFI	TLI	RMSEA	RMSEA IC 90%	
									Lower	Upper
Model of 1 factor	E1 COL	70	4076.291	77	.00	.825	.793	.239	.233	.245
Model of 3 factors	E1 COL	73	533.037	74	.00	.980	.975	.083	.076	.089
Model of 1 factor	E2 COL	70	1725.995	77	.00	.733	.685	.223	.214	.232
Model of 3 factors	E2 COL	73	223.692	74	.00	.976	.970	.069	.058	.079
Model of 1 factor	E2 PER	70	1542.098	.77	.00	.836	.806	.221	.211	.230
Model of 3 factors	E2 PER	73	238.993	74	.00	.982	.977	.076	.065	.086

*E1* study 1, *E2* study 2, *COL* Colombian sample, *PER* Peruvian sample, *EBEA* Brief Acculturative Stress Scale, *Par* number of parameters in the model, *χ*^*2*^ chi-square, *DF* degrees of freedom, *CFI* comparative fit index, *TLI* Tucker-Lewis Index, *RMSEA* root mean square error of approximation

The factor loads, factor covariances, and reliability estimates for each dimension are presented in Table 4. As can be seen, the factorial saturations of each of the dimensions have high (λ > .5) and statistically significant factorial loads in all models. As for the representation of the relationships between factors, the dimensions showed moderate (*r* > .30) to large (*r* > .50) correlations (Cohen, 1988) in both studies. Finally, reliability estimates were higher than .80, demonstrating high levels of internal consistency in all dimensions and in the samples of both studies (see Table 4).

**Table 4:** Standardised factorial saturations and reliability estimates (Cronbach’s alpha and omega coefficient) of the EBEA in both samples

Brief Acculturation Stress Scale (EBEA)	E1 COL			E2 COL			E2 PER		
	PSP	PSE	ASR	PSP	PSE	ASR	PSP	PSE	ASR
Preparation and departure from country of origin (PSP)									
1. Prepare to leave your country.	.81*			.84*			85*		
2. Get the money to leave.	.76*			.77*			77*		
3. Leave your country.	.85*			.90*			90*		
4. Moving from your country to Chile.	.81*			.84*			84*		
5. Having to be separated from your family.	.76*			.79*			79*		
6. Stop seeing friends.	.72*			.67*			67*		
Socioeconomic concerns (PES)									
1. Cover your basic needs.		.88*			.85*			85*	
2. Find a place to live.		.89*			.86*			86*	
3. Find a job.		.87*			.90*			90*	
4. Stabilise economically.		.86*			.86*			87*	
Adaptation to the recipient society (ASR)									
1. Adapt to Chilean’s way of speaking.			.84*			.75*			76*
2. Initiate contact with the neighbours.			.92*			.89*			89*
3. Make friends.			.95*			.90*			90*
4. Establish relationships with the people you work with.			.89*			.87*			87*
**Covariate factors**									
Socioeconomic concerns (PES)	.66*			.42*			.74*		
Adaptation to the recipient society (ASR)	.43*	.54*		.28*	.42*		.48*	.53*	
**Reliability estimates**									
Alpha (α)	.87	.90	.92	.86	.86	.83	.88	.89	.86
Omega (ω)	.87	.90	.92	.86	.86	.83	.88	.89	.86

*E1* study 1, *E2* study 2, *COL* Colombian sample, *PER* Peruvian sample, *EBEA* Acculturation Stress Brief Scale, *PSP* preparation and departure from the country of origin, *PSE* socioeconomic concerns, *ASR* adaptation to the receiving society

*p < .001

### Evidence of factorial invariance

The results of the fit indexes on the analysis by sex are presented in Table 5 (study 1). As can be seen, CFI and RMSEA fit indexes do not show statistically significant differences in fit between metric or scalar model, compared with configural model. These results show that the scores for each of the dimensions of EBEA are equivalent between men and women.

**Table 5:** Goodness-of-fit indexes of nested model of factor invariance analysis according to sex

	*χ*^2^	*DF*	*p*	RMSEA	CFI	Δ_χ2_	Δ_*DF*_	Δ_*p*_	Δ_RMSEA_	Δ_CFI_
Configural	637.525	173	.000	.077	.980					
Metric	581.898	187	.000	.068	.983	13.042	14	.523	− .009	.003
Scalar	559.783	201	.000	.063	.984	30.297	14	.006	− .014	.004

Table 6 shows the results of measurement invariance testing, between Colombians and Peruvians (study 2). Again, EBEA shows an excellent fit, without statistically significant differences being detected between the different levels of invariance. These results show that the scores of the different dimensions are equivalent between migrants from Colombia and Peru.

**Table 6:** Goodness-of-fit indexes of nested model of factor invariance analysis according to home country

	*χ*^2^	*DF*	*p*	RMSEA	CFI	Δ_χ2_	Δ_*DF*_	Δ_*p*_	Δ_RMSEA_	Δ_CFI_
Configural	504.480	173	.000	.068	.978					
Metric	515.856	187	.000	.065	.979	30.543	14	.006	− .003	.001
Scalar	534.083	201	.000	.063	.978	43.845	14	.000	− .005	.000

In summary, the results of the factorial invariance analyses of both studies show that it can be assumed measurement equivalence between men and women (study 1), and between people from Colombia and Peru living in Chile (study 2).

### Evidence of nomological validity

In order to present evidence of nomological validity, structural equation models (M1, M2, and M3) were estimated, and the dimensions of the EBEA with the BAI (study 1: M1) and the SPS (study 2: M2, Colombian sample; M3, Peruvian sample).

Table 7 shows the global adjustment indicators of models M1, M2, and M3. The estimated goodness-of-fit indexes of the models indicate that they were good representations of the observed relationships.

**Table 7:** Global adjustment indicators for models M1, M2, and M3

Models	Par	*χ*^2^	*DF*	*p*	CFI	TLI	RMSEA	RMSEA IC 90%	
								Lower	Upper
M1	160	1788.810	554	.00	.975	.973	.049	.047	.052
M2	106	387.148	164	.00	.968	.963	.056	.049	.063
M3	106	423.231	164	.00	.974	.970	.063	.056	.071

*M1* model 1, *M2* model 2, *M3* model 3, *Par* number of model parameters, *χ*^*2*^ chi-square, *DF* degrees of freedom, *CFI* comparative fit index, *TLI* Tucker-Lewis Index, *RMSEA* root mean square error of approximation

Finally, Table 8 shows the relations between the dimensions of the EBEA with the BAI (study 1) and the SPS (study 2). In study 1, the BAI presented positive correlations of small magnitudes (*r* > .10) (Cohen, 1988) with the three dimensions of the EBEA: preparation and departure from the country of origin (*ρ* = .117), socioeconomic concerns (*ρ* = .105), and adaptation to the receiving society (*ρ* = .200). In study 2, the SPS presented negative correlations of small magnitudes (*r* > .10) with the three dimensions of the EBEA in both samples (see Table 8).

**Table 8:** Standardised poly-correlation matrix

	Variables	PSP	PSE	ASR
Study 1	BAI	.117*	.105*	.200**
Study 2	SPS COL	− .246**	− .246**	− .193**
	SPS PER	− .275**	− .272**	− .125*

*COL* Colombian sample, *PER* Peruvian sample, *BAI* Beck’s Anxiety Inventory, *SPS* psychological health, *PSP* preparation and departure from the country of origin, *PSE* socioeconomic concerns, *ASR* adaptation to the recipient society

*p < .01

**p < .001

## Discussion

This article aimed to provide the first confirmed evidence of the validity and reliability of the scores from the abbreviated measure of acculturation stress in migrant populations. These findings are beneficial for rapid screening of the effects of acculturation stress in migrant populations.

To provide evidence of the scores’ validity, we examined the factorial structure of the scale and the degree of relationship, with other variables theoretically related to anxiety and psychological health in two different studies. The results in both studies indicated the scale presents a factorial structure of three dimensions: (1) stress derived from preparation and departure from the country of origin, (2) stress produced by socioeconomic concerns in the host country, and (3) typical tensions of adaptation to sociocultural changes or Chilean society. Likewise, according to Chen (2007) invariance testing suggested standards, the results show that the model of three factors of the EBEA is equivalent between men and women, as well as between Colombians and Peruvians, which suggests that comparisons between groups based on these variables (sex and home country) are possible to evaluate.

These dimensions were consistent with those reported in the literature on stress-related factors (Bekteshi & Kang, 2020). Although acculturation is a process immigrants experience once they arrive in the host country, the stress it can cause manifests itself from the moment the change of country is planned (Ugalde-Watson et al., 2011). Thinking about how to adapt to the new culture, how to generate new social networks, and how to maintain a favourable economic status in the new country, leaving behind one’s culture, family, and loved ones, and preparing to leave is one of the most stressful stages of acculturation (Ugalde-Watson et al., 2011; Urzúa, Basabe, et al., 2017). However, meeting basic needs, finding work, and a place to live are part of the socioeconomic concerns that affect the migrant population the most (Bekteshi & Kang, 2020; Ugalde-Watson et al., 2011), because many times people migrate without sufficient support networks in the host country (de Haymes, Martone, Muñoz, & Grossman, 2011). From this point on, the migrant is often left in a more vulnerable social position and with few opportunities to get a stable job to maintain the personal-family economy as opposed to local residents (Urzúa et al., 2016). Finally, cultural differences may strain the process of sociocultural adaptation, as the new environment will demand adaptation experiences that may exceed people’s resources and capacities to cope with these demands (Bekteshi & Kang, 2020; Urzúa et al., 2016). Some authors have shown that the perception of large cultural differences correlates with higher levels of acculturation stress among migrants (Urzúa, Basabe, et al., 2017).

Regarding its nomological validity, study 1 indicated that acculturation stress was positively related to the Columbian’s anxiety levels. Study 2 indicated that the higher the levels of acculturation stress, the lower the scores on the psychological health scale answered by both Colombians and Peruvians. Although the correlations with the other instruments were small, some authors noted there were cases where the relationships between dimensions could have important consequences if they refer to recurrent events over time (Funder & Ozer, 2019). In the case of both studies, perceiving constant stress from living in a new culture could accumulate seemingly small anxious effects, but have important implications for the psychological health, well-being, and quality of life of immigrants in Chile. Additionally, the low magnitude of the correlations can be interpreted as an indicator that they were linked constructs, but theoretically different. This provides evidence that acculturation stress is a type of stress specific to the migratory context.

For internal consistency, the alpha and omega coefficients were calculated for each of the scale dimensions in each study sample. The coefficients estimated in all the analyses were excellent, indicating that the scores on the abbreviated acculturation stress scale are reliable. These results caught our attention because, in general, short scales tend to present less reliable results than scales with a greater number of items.

As this was the first approach to the construction of a new instrument, some limitations of the study should be mentioned. First, the research was of a transversal nature, so no causalities can be assumed between the relationships of the variables used in the studies. Second, given the difficulty of obtaining a valid sample universe, the sample was non-probabilistic; therefore, the results could not reflect with certainty the experiences of migrants who do not go to places with a common migrant population, like the places where they were surveyed. Finally, the migrants who participated in this study were from only two Latin American countries (Colombia and Peru) and resided in three cities in Chile (Arica, Antofagasta, and Santiago), so it is necessary to continue exploring the behaviour of the scale among migrants from other Spanish-speaking nations. Despite this, it was noted that another advantage of having a valid instrument is being able to open other lines of study, such as the mediating effect of residence time, or other variables, in the relationship between acculturation stress and some variables of psychological health or well-being.

## Conclusions

The EBEA managed to adequately represent its structure of three latent factors and demonstrated valid and reliable scores for its use in migrant populations equivalent to those of the samples examined in the present study. In addition, EBEA demonstrated factor invariance for the comparison of scores between groups of male and female, and Colombians and Peruvians. Finally, the EBEA presented a measure that offered a fast and useful screening tool for researchers who needed a brief measure for investigations in the area. Although it is necessary to continue exploring the psychometric properties of the scale among other migrant groups, actually EBEA is a tool that can contribute to the work of education, prevention, and intervention in the field of general health and migrants’ mental health.

## Data Availability

The data used and analysed during the current study are not publicly available due ethics privacy of participants but are available from the corresponding author on reasonable request.

## Abbreviations

COVID-19: Coronavirus disease
EBEA: Escala Breve para la Evaluación del Estrés por Aculturación, Brief Scale of Stress by Acculturation
BAI: Beck Anxiety Inventory
SPS: Psychological health
WLSMV: Weighted least square mean and variance
CFI: Comparative fit index
TLI: Tucker-Lewis Index
RMSEA: Root mean square error of approximation
PSP: Preparation and departure from the country of origin
PES: Socioeconomic concerns
ASR: Adaptation to the recipient society
E1: Study 1
E2: Study 2
